# Case Report: Paralytic Ileus: A Potential Extrapulmonary Manifestation of Severe COVID-19

**DOI:** 10.4269/ajtmh.20-0894

**Published:** 2020-08-31

**Authors:** Yassmin S. Ibrahim, Gowri Karuppasamy, Jessiya V. Parambil, Hussam Alsoub, Shaikha D. Al-Shokri

**Affiliations:** 1 Internal Medicine Department, Hamad Medical Corporation, Doha, Qatar;; 2 Infectious Disease Department, Hamad Medical Corporation, Doha, Qatar

## Abstract

The COVID-19 pandemic has recently spread worldwide, presenting primarily in the form of pneumonia or other respiratory disease. In addition, gastrointestinal manifestations have increasingly been reported as one of the extrapulmonary features of the virus. We report two cases of SARS-CoV-2 infection complicated by paralytic ileus. The first patient was a 33-year-old man who was hospitalized with severe COVID-19 pneumonia requiring ventilator support and intensive care. He developed large bowel dilatation and perforation of the mid-transverse colon, and underwent laparotomy and colonic resection. Histopathology of the resected bowel specimen showed acute inflammation, necrosis, and hemorrhage, supporting a role for COVID-19–induced micro-thrombosis leading to perforation. The second patient was a 33-year-old man who had severe COVID-19 pneumonia, renal failure, and acute pancreatitis. His hospital course was complicated with paralytic ileus, and he improved with conservative management. Both cases were observed to have elevated liver transaminases, which is consistent with other studies. Several authors have postulated that the angiotensin-converting enzyme 2 receptors, the host receptors for COVID-19, that are present on enterocytes in both the small and large bowel might mediate viral entry and resultant inflammation. This is a potential mechanism of paralytic ileus in cases of severe COVID-19 infection. Recognizing paralytic ileus as a possible complication necessitates timely diagnosis and management.

## INTRODUCTION

COVID-19, the disease caused by SARS-CoV-2, was initially identified as a cluster of pneumonia cases in Wuhan, a city in Hubei, China, toward the end of 2019.^1^ Several extrapulmonary features have been reported for this disease. Studies have estimated that 30–40% of cases are asymptomatic, irrespective of the viral load.^2,3^ Gastrointestinal symptoms have been reported to occur in 17.6% of infected patients.^4^ The most common symptoms documented are loss of appetite, followed by diarrhea, nausea/vomiting, and abdominal pain.^4^ Abnormal liver biochemical tests and fecal shedding of the virus have also been observed.^4–6^ However, there is scarcity of knowledge in the implication of this infection on gastrointestinal complications. We describe two cases of severe COVID-19 pneumonia who developed paralytic ileus during their disease course, which may represent one of the luminal manifestations of severe SARS-CoV-2 infection.

## CASE DESCRIPTIONS

The first case was a 33-year-old man who presented with a 2-day history of fever, cough, and sore throat. Initial laboratory investigations were normal apart from elevated inflammatory markers. Chest X-ray showed haziness along the mid and lower lung zones, leading to suspicion of viral pneumonia, and his nasopharyngeal swab reverse transcriptase–PCR (RT-PCR) tested positive for SARS-CoV-2. Two days after admission, he developed tachypnea and desaturation with the progression of infiltrates on chest imaging. He was intubated, received vasopressor support, and admitted to intensive care. The patient received COVID-19 treatment (ceftriaxone, azithromycin, hydroxychloroquine, methylprednisolone, and a dose of tocilizumab) guided by the local protocol.

**Figure 1.**
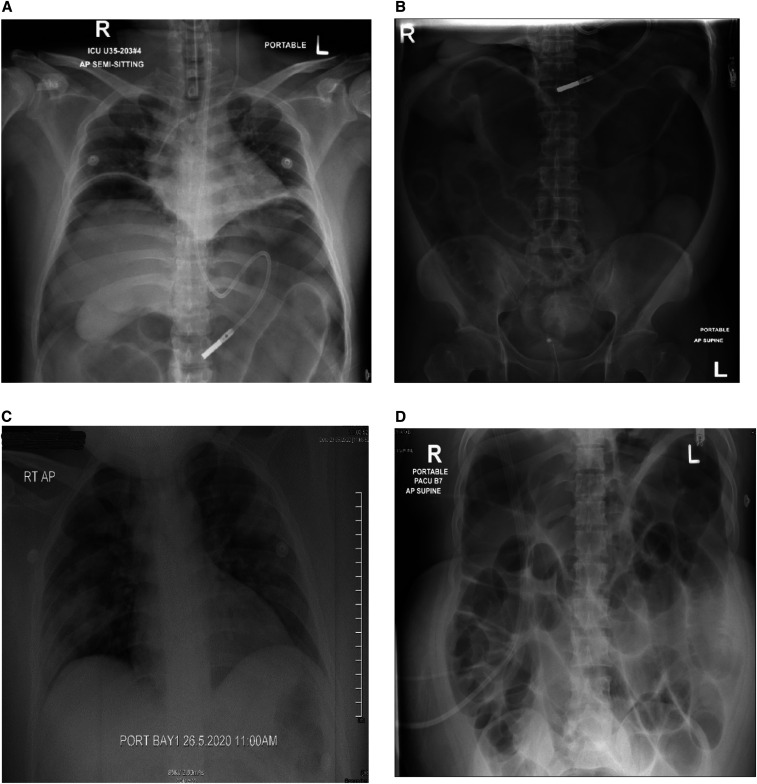
Radiographs for patients diagnosed with COVID-19 pneumonia. (**A**) Chest radiograph for case 1 showing bilateral free air under the diaphragm and (**B**) abdominal radiograph showing distended bowel loops. (**C**) Chest radiograph for case 2 showing bilateral patchy infiltrates and (**D**) abdominal radiograph showing diffuse dilatation of the small and large bowel loops.

**Figure 2.**
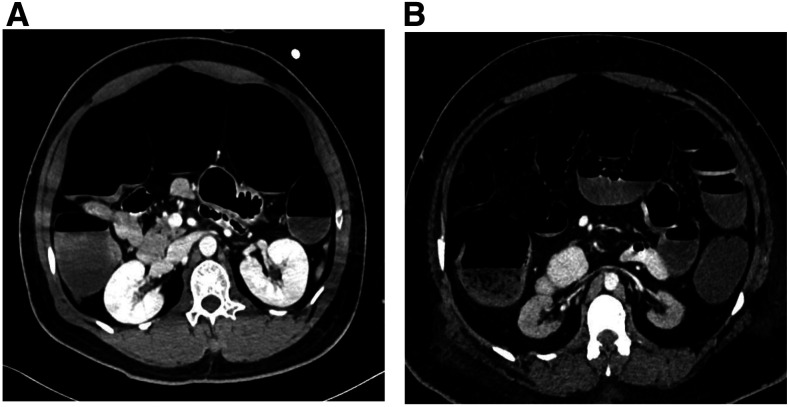
Computed tomography (CT) scan of the abdomen in patients diagnosed with COVID-19 pneumonia. (**A**) Contrast-enhanced CT scan, an axial plane of the abdomen of the first case showing large bowel dilatation with no stricture or obstruction, and patent mesenteric vessels. (**B**) Contrast-enhanced CT scan, an axial plane of the abdomen of the second case showing diffusely distended small and large bowel with air–fluid levels.

The patient was extubated after 4 days but was re-intubated because of increased oxygen requirements. Examination revealed distended and tense abdomen with epigastric tenderness, exaggerated bowel sounds, and empty rectum. He was noted to have abnormal liver enzymes, alanine aminotransferase (ALT) was 1,589 U/L, and aspartate aminotransferase (AST) was 1,856 U/L. Abdominal X-ray showed bilateral free air under the diaphragm and distended bowel loops (Figure 1A and B). Computed tomography (CT) scan of the abdomen showed large bowel dilatation from the cecum down to the rectum with no stricture or transitional area of obstruction, and patent mesenteric vessels (Figure 2A). Surgical evaluation was sought, and emergency laparotomy was performed. The operative findings were pyogenic membranes and purulent fluid in the peritoneal cavity and a 2-mm perforation in the anterior aspect of the mid-transverse colon with three serosal tears of 3–4 cm distal to the perforation; the rest of the bowel was healthy. He underwent primary repair of the perforated colon and lavage. His condition improved, and he was gradually introduced to enteral feeding. Liver enzymes improved over the next week. However, 10 days later, the patient again developed respiratory distress, and repeat CT abdomen showed significant free peritoneal air with fluid contrast layering at the rectovesical pouch, raising suspicion of extra-luminal bowel contrast leak. No bowel dilatation was found. Urgent laparotomy revealed a localized leak of the repaired perforated transverse colon; transverse loop colostomy was performed. The patient’s hospital course was also complicated by renal failure, which was attributed to acute tubular necrosis secondary to infection and bowel perforation. He remains hospitalized in intensive care at the time of writing this report.

The second case was a 33-year-old man who was brought to the hospital in severe respiratory distress. He had abdominal pain; examination revealed epigastric and right hypochondrial tenderness with guarding. His laboratory evaluation showed leukocytosis, elevated inflammatory markers, and impaired renal function with blood urea of 55.78 mmol/L, creatinine 1,891 µmol/L, and bicarbonate 1.9 mmol/L. Other significant investigations included lipase of > 1,200 U/L, amylase 390 U/L, ALT 187 U/L, and AST 125 U/L. He was noted to have deranged electrolytes, with potassium of 2.2 mmol/L and calcium 0.96 mmol/L. His nasopharyngeal RT-PCR was positive for SARS-CoV-2. Bilateral pneumonic patches were observed on the chest X-ray (Figure 1C). He was intubated and urgent hemodialysis was initiated, and he was admitted to the intensive care unit as a case of severe COVID-19 pneumonia, renal failure, and acute pancreatitis. Ultrasound abdomen revealed healthy gallbladder but noted dilated fluid-filled bowel loops in the left lower abdomen. He was extubated on day 5 of admission. Two days after extubation, the patient developed bilious vomiting and abdominal discomfort. He did not have bowel movements for the preceding 4 days but was passing flatus. On examination, he had a distended abdomen with no guarding, but bowel sounds were sluggish. Abdominal X-ray showing diffuse dilatation of the small and large bowel loops (Figure 1D). Computed tomography scan of the abdomen showed diffusely distended large bowel, from the anus to the cecum, filled with fluid, and air–fluid levels extending to involve the small bowel proximally up to the third part of the duodenum. There was no transitional zone of narrowing, mass, or bowel wall thickening. Minimal peripancreatic fat stranding was noted with no fluid collection. Both kidneys were small in size, and an incidental finding of soft tissue lesion at the right upper pole was noted (Figure 2B). He was evaluated by the surgery team, who advised conservative management with a nasogastric tube insertion and correction of electrolytes. The patient was kept nil per oral, and total parenteral nutrition was initiated. He received prokinetic agents and intravenous potassium replacement to maintain target serum potassium of 4 mmol/L. The patient’s condition improved over the next week, and he was able to tolerate an oral diet. A repeat CT scan performed for further evaluation of renal mass showed improvement in the significant bowel dilatation and resolution of small bowel dilatation. The patient is currently on renal replacement therapy three times a week through a permanent subclavian tunneled catheter.

## DISCUSSION

Gastrointestinal involvement has been increasingly reported in association with the SARS-CoV-2 infection. A multicenter cohort study of hospitalized U.S. adults found that two-thirds of patients with COVID-19 had at least one gastrointestinal symptom.^7^ A review of 29 studies noted that 12% of patients with SARS-CoV-2 infection had gastrointestinal symptoms, including diarrhea, nausea, and vomiting.^8^ The clinical significance of this presentation was demonstrated in a review by Mao et al.,^9^ who reported that patients who presented with gastrointestinal system involvement had delayed diagnosis and also tended to have a poorer disease course.

It has been proposed that the angiotensin-converting enzyme 2 (ACE2) receptor plays a central role in the mechanism of gastrointestinal tract involvement in COVID-19. The ACE-2 receptor is the functional host receptor for the SARS-CoV-2 virus.^10,11^ Although ACE2 is highly expressed in alveolar cells in the lungs, ACE2 receptors are also abundant in the gastrointestinal tract, especially in the small and large intestines.^12,13^ The gastrointestinal symptoms that appear early during COVID-19 suggest that the small bowel may be an important entry site for the virus.^14^ Zhang et al.^15^ postulated that ACE2 expression on small intestinal enterocytes may mediate the invasion of the virus and activation of gastrointestinal inflammation. Therefore, this could be a potential mechanism of paralytic ileus in severe COVID-19 cases.

There is a growing body of evidence describing large bowel involvement in COVID-19 infection. Carvalho et al.^16^ described a case of SARS-CoV-2 gastrointestinal infection causing acute hemorrhagic colitis with endoscopic findings of coloproctopathy but normal histologic findings. Sattar et al.^17^ reported three cases of COVID-19 with colonic findings, including colitis and air in the bowel wall.

However, there are few reports of paralytic ileus in patients with COVID-19. Kaafarani et al.^18^ described a series of 141 critically ill patients with COVID-19, where half of the patients developed hypomotility-related complications of variable severity. Only two patients were diagnosed with a colonic paralytic ileus (Ogilvie’s syndrome). Similarly, the patient in case 1 had extensive large bowel dilatation and mid-transverse colon perforation. Histopathology of the resected bowel specimen showed fat necrosis, acute inflammation, reactive fibroblastic proliferation, and hemorrhage. However, the mesenteric vessels were patent on imaging, suggesting that the etiology could be SARS-CoV-2–induced micro-thrombosis. It has been hypothesized that SARS-CoV-2 causes vascular endothelial cell inflammation, leading to impaired microcirculatory function in different vascular beds.^19^

Moreover, Bhayana et al.^20^ reported bowel wall abnormalities in 31% of abdominal CT scan images in COVID-19 patients, including pneumatosis and portal venous gas. The patients were found to have bowel infarction due to ischemic enteritis with patchy necrosis and fibrin thrombi in the arterioles. Our second case had diffuse distension of both small bowel and large bowel but responded well to conservative management. The development of ileus, in this case, may also be explained by electrolyte derangement, as the patient had hypokalemia and hypocalcemia. However, SARS-CoV-2 may cause ileus in an independent mechanism, as alluded previously.

Our cases were both observed to have elevated liver transaminases. In a systematic review, abnormal liver enzyme levels were noted in 15–20% of patients.^11^ Studies by Mao et al.^9^ and Guan et al.^21^ found that patients with severe COVID-19 had higher rates of abnormal liver function.^12,21^

Case 2 developed severe acute pancreatitis of unclear etiology, with elevated lipase and peripancreatic fat stranding on imaging. A few similar cases have been reported, but there is uncertainty about its pathogenesis.^22,23^ Theories include acute inflammation associated with the expression of ACE2 in the pancreas and heightened systemic inflammatory response from cytokine storm syndrome leading to multi-organ dysfunction.^24^

A review by Bourgonje et al.^25^ also points out that it is the relative proportion of ACE to ACE2 receptors that is responsible for the resultant pro-inflammatory and pro-fibrotic symptomatology, and it has been reported that these may be affected by gender, with males having a relatively higher proportion of ACE to ACE2, thus favoring inflammation and colitis. Both our patients were male, supporting this theory. Further studies are needed to elaborate on the causative relationship between SARS-CoV-2 and the gastrointestinal manifestations of COVID-19.

In conclusion, we report paralytic small and large bowel ileus as a complication of COVID-19. Furthermore, we explore a potential mechanism of ileus and explore the management strategies. The added value of the present case report is the detailed histopathological evidence supporting a role for COVID-19–induced micro-thrombosis, thereby compromising microcirculatory function and resultant colonic bowel dilatation and perforation in the first patient. Recognizing paralytic ileus as a possible complication necessitates timely diagnosis and management.
